# Unusual Etiology of Chronic Cough and Syncope as Chiari Malformation Type 1

**DOI:** 10.7759/cureus.40598

**Published:** 2023-06-18

**Authors:** Kunj Patel, Kashmira Wani, Amir Daneshvar, Jasmine Omar

**Affiliations:** 1 Internal Medicine, Henry Ford Health System, Detroit, USA

**Keywords:** laminectomy, mri brain, syncope, chiari 1 malformation, chronic cough

## Abstract

Chronic cough is a common chief complaint in ambulatory clinics. Unlike most cases that are caused by upper airway cough syndrome, gastroesophageal reflux disease, asthma, and non-asthmatic eosinophilic bronchitis, chronic cough can also be the presenting feature of a Chiari malformation. Our case is that of a 39-year-old female who had a chronic cough associated with shortness of breath, and when severe, associated with loss of consciousness. Her cough was refractory to conventional management. Further workup including pulmonary functions tests (PFT), laryngoscopy, high-resolution CT of the chest, an upper GI series, and esophageal pH manometry study were all normal. An MRI of her brain was obtained due to her syncopal episodes and revealed findings concerning a type 1 Chiari malformation. She subsequently underwent a Chiari decompression with patchy duraplasty and tonsilloplasty with cervical vertebrae 1 and 2 (C1-C2) laminectomy with a resolution of her symptoms. Chiari malformations are sometimes inherited but are often sporadic in nature, and, thus, appropriate diagnosis is key. Our patient is unique in that she presented at an older age, suggesting that atypical etiologies of a chronic cough refractory to conventional treatments must be considered.

## Introduction

Chronic cough lasting longer than eight weeks is a common presentation seen in an ambulatory setting [1]. In adults with normal lung physiology, most cases are due to one of four conditions: upper airway cough syndrome, gastroesophageal reflux disease, asthma, and nonasthmatic eosinophilic bronchitis [1]. Chiari malformations are a spectrum of anatomic anomalies of the craniocervical junction with downward displacement of the cerebellar structures [2]. They are primarily classified into three groups. Chiari malformation type 1 is the most common and is typically defined by abnormally shaped cerebellar tonsils displaced below the level of the foramen magnum and presents in the first few decades of a patient’s life [2]. Here we report an unusual case of a 39-year-old female with chronic cough as a unique presenting symptom of Chiari malformation type 1 at an atypical age.

## Case presentation

A 39-year-old female with a past medical history of iron deficiency anemia presented to the clinic with symptoms of chronic cough for five years duration. The cough is nonproductive and persistent throughout the day even at rest and without any sputum production. Occasionally, she reported coughing at night which caused her to awaken. Her symptoms improved when she was on a liquid diet but otherwise, there were no known alleviating factors. She only developed associated shortness of breath if she had a coughing spell for a prolonged period of time but otherwise denied dyspnea. Her most severe coughing lasted for two minutes followed by a burning-like sensation in her head and numbness in her fingers followed by sudden loss of consciousness. She otherwise denied any sinus congestion, fever, chest pain, palpitations, difficulty swallowing, nausea, vomiting, reflux, epigastric pain, leg swelling, or orthopnea. She denied tobacco use, marijuana use, alcohol use, or vaping history. She did not use any medications on a daily basis.

**Figure 1:**
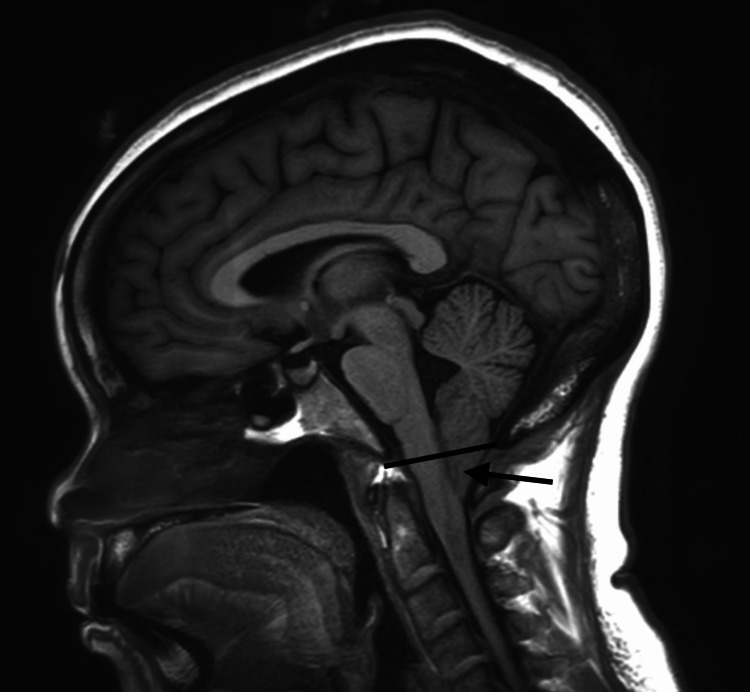
Sagittal T1-Weighted MRI brain showing 15.5 mm tonsillar protrusion (arrow)

Her vital signs and physical exam were unremarkable. Her lab work including a complete metabolic panel and complete blood count was unremarkable. Over the next several months, she was trialed on multiple medications, initially with oral cetirizine and fluticasone nasal spray then esomeprazole for eight weeks, followed by inhaled steroids and albuterol, without improvement in her symptoms. She was seen by otolaryngology and found to have a normal laryngoscopy. Pulmonology completed a pulmonary function test which revealed a forced vital capacity (FVC) of 2.68 L (95% of reference), forced expiratory volume (FEV1) of 2.12 L (90% of reference), and an FEV1/FVC ratio of 79% suggestive of a normal spirogram. A methacholine challenge was negative. Given her symptomatic improvement when on a liquid-based diet, there was a concern for oropharyngeal dysphagia, and she was seen by speech-language pathology and allergy. However, both a swallow evaluation and a skin prick test with 25 antigens were negative. A high-resolution CT chest showed no evidence of interstitial lung disease or air trapping. Due to her syncopal episodes, she completed a cardiac and neurologic workup, including a 14-day Holter monitor, transthoracic echocardiogram, electroencephalogram, CT head, and CTA head and neck which were all unremarkable. Over the next six months, her syncopal episodes began to increase in frequency, and she began to develop episodes even without coughing. An MRI brain with and without contrast revealed a 15.5 mm tonsillar ectopy with the fullness of the foramen magnum with peg-shaped cerebellar tonsil concerning Chiari 1 malformation (Figure 1).

**Figure 2:**
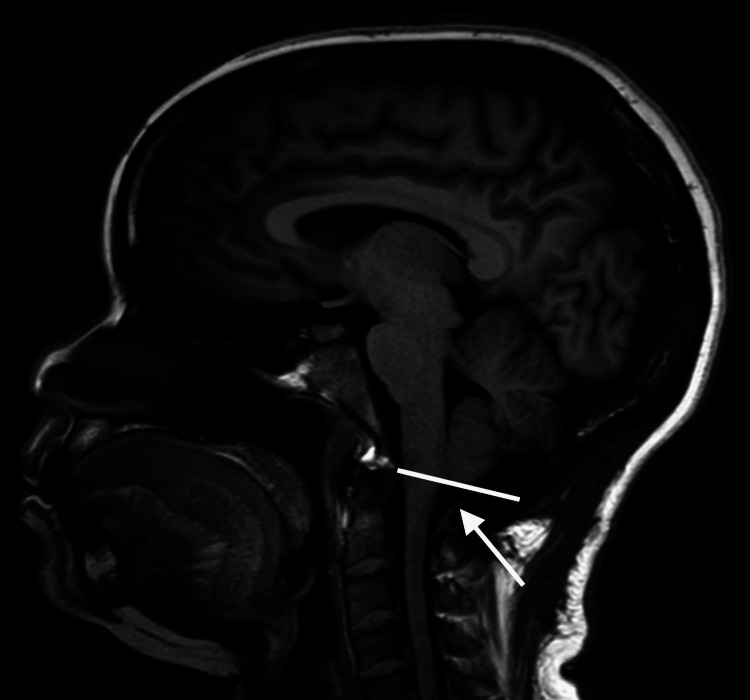
Post-surgical sagittal T1-Weighted MRI brain without tonsillar protrusion

She was seen by neurosurgery and underwent a Chiari decompression with patchy duraplasty and tonsilloplasty with C1-2 laminectomy. Following her procedure, she had complete resolution of her cough and no further syncopal episodes up to two years post-operation. Her initial follow-up MRI obtained two months later is shown below (Figure 2).

## Discussion

Chiari malformations are a rare set of congenital conditions defined by anatomic anomalies involving the craniocervical junction with downward displacement of cerebellar structures [3]. These disorders are classified into four groups, the most common of which is a type I malformation involving abnormal cerebellar tonsils that are displaced below the foramen magnum. In a type II malformation, there is downward displacement of the cerebellar vermis and tonsils and evidence of spinal myelomeningocele. A type III malformation is particularly rare and involves a cervical or occipital encephalocele with a displacement of cerebellar structures into the encephalocele. Chiari type IV malformations are cerebellar hypoplasia that is not related to other Chiari malformations [3].

The pathophysiology of Chiari malformations is not fully understood; however, various theories postulate that the malformations may be due to genetic defects in the development of the hindbrain [3], while others believe that fetal hydrocephalus causes compression of the cerebellum and brainstem [4]. There are alternative theories such as Oldfield's piston theory where propagation of the syringomyelia is based on the cardiac cycle [5]. In this theory, when there is an obstruction at the region of the foramen magnum, the rapid to and fro movement of the cerebral spinal fluid causes abrupt piston-like caudal movement of the tonsils [5]. However, this theory certainly does not explain the initial obstruction.

While the true prevalence of Chiari malformations is unclear, there has been an increasing incidental detection noted with the use of MRI imaging modalities; 1-3.6% of MRIs have detected the presence of a Chiari malformation [6]. Prior to the widespread use of MRI imaging, Chiari malformations were believed to be a disorder seen in adolescents; however, asymptomatic patients are now being diagnosed during childhood through incidental findings on MRI [7].

The clinical manifestations of Chiari malformations vary from asymptomatic to severe neurological dysfunction. Cerebellar symptoms including ataxia and nystagmus can be seen [7]. Other symptoms include voice hoarseness, vocal cord paralysis, dysarthria, palatal weakness, pharyngeal achalasia, tongue atrophy, recurrent aspiration, and visual nystagmus [3,8]. Paroxysmal occipital headaches are often the main symptoms seen in adults; the headaches are often worsened with coughs or laughter [6]. In the event of brainstem compression, symptoms may manifest as weakness or hyperreflexia.

The diagnosis of Chiari malformation is best made through the use of an MRI of the brain and spinal cord to assess for the displacement of the cerebellum/brain stem and evidence of hydrosyringomyelia [9]. For patients who are not ideal candidates for MRI, a CT scan can be used as an alternative imaging modality. The formal radiographic diagnosis of a type 1 Chiari malformation is made when either of the cerebellar tonsils is displaced by 5 mm or more below the foramen magnum [9].

The management of a type 1 Chiari malformation depends on the severity of symptoms and the presence of anatomical abnormalities such as syringomyelia or CSF obstruction. For asymptomatic patients, conservative management with MRI surveillance is recommended. For patients who have syringomyelia or CSF flow obstruction but no neurological symptoms, spontaneous resolution of the syringomyelia has been seen, and, therefore, conservative management remains a valid option [10]. Posterior fossa decompressive surgery is indicated for patients with evidence of complete CSF flow obstruction on imaging, as well as for patients with symptoms including myelopathy, cough-induced headaches, and cerebellar deficits. The purpose of surgery is to restore normal CSF flow in the foramen magnum [11]. The majority of patients who undergo decompressive surgery experience improvement in their neurological symptoms postoperatively.

Our patient had an atypical presentation of a type 1 Chiari malformation, as her initial symptom onset was in her mid-thirties, older than the standard onset of symptoms seen in children and adolescents. Her main initial symptom included a five-year history of cough associated with headaches and syncopal episodes. She underwent an extensive cardiopulmonary assessment of her chronic coughs with largely unremarkable findings. However, her Chiari malformation was able to be detected through the use of MRI brain imaging, and her symptoms resolved after definitive neurosurgery. There are some uncertainties within this case. In particular, the patient had nocturnal symptoms, cough, and possible improvement with a liquid diet which could raise the concern for esophageal webs or Plummer-Vinson syndrome. However, a formal upper endoscopy was unable to be obtained as she began to develop syncopal episodes, and her work-up focused more on neurological etiologies.

## Conclusions

Chiari type 1 malformations are an incredibly rare etiology of chronic cough. Though they are infrequent, it is important to specifically suspect this pathology when a patient’s cough is associated with neurological symptoms. With appropriate diagnosis, this disease can be managed effectively with a good prognosis as seen in our patient.
